# Comparative Insights into Photosynthetic, Biochemical, and Ultrastructural Mechanisms in Hibiscus and Pelargonium Plants

**DOI:** 10.3390/plants13192831

**Published:** 2024-10-09

**Authors:** Renan Falcioni, Werner Camargos Antunes, Roney Berti de Oliveira, Marcelo Luiz Chicati, José Alexandre M. Demattê, Marcos Rafael Nanni

**Affiliations:** 1 Department of Agronomy, State University of Maringá, Av. Colombo, 5790, Maringá 87020-900, Paraná, Brazil; wcantunes@uem.br (W.C.A.); rboliveira@uem.br (R.B.d.O.); mlchicati@uem.br (M.L.C.); mrnanni@uem.br (M.R.N.); 2 Department of Soil Science, Luiz de Queiroz College of Agriculture, University of São Paulo, Av. Pádua Dias, 11, Piracicaba 13418-260, São Paulo, Brazil; jamdemat@usp.br

**Keywords:** biochemical compounds, chloroplasts, chlorophyll a fluorescence, gas exchange analyser, horticulturae, hyperspectroscopy, microscopies, mitochondria, plant breeding

## Abstract

Understanding photosynthetic mechanisms in different plant species is crucial for advancing agricultural productivity and ecological restoration. This study presents a detailed physiological and ultrastructural comparison of photosynthetic mechanisms between Hibiscus (*Hibiscus rosa-sinensis* L.) and Pelargonium (*Pelargonium zonale* (L.) L’Hér. Ex Aiton) plants. The data collection encompassed daily photosynthetic profiles, responses to light and CO_2_, leaf optical properties, fluorescence data (OJIP transients), biochemical analyses, and anatomical observations. The findings reveal distinct morphological, optical, and biochemical adaptations between the two species. These adaptations were associated with differences in photochemical (*A*_MAX_, *E*, *C*_i_, *i*WUE, and α) and carboxylative parameters (*VC*_MAX_, ΓCO_2_, *g*_s_, *g*_m_, *C*c, and *AJ*_MAX_), along with variations in fluorescence and concentrations of chlorophylls and carotenoids. Such factors modulate the efficiency of photosynthesis. Energy dissipation mechanisms, including thermal and fluorescence pathways (ΦPSII, ETR, NPQ), and JIP test-derived metrics highlighted differences in electron transport, particularly between PSII and PSI. At the ultrastructural level, Hibiscus exhibited optimised cellular and chloroplast architecture, characterised by increased chloroplast density and robust grana structures. In contrast, Pelargonium displayed suboptimal photosynthetic parameters, possibly due to reduced thylakoid counts and a higher proportion of mitochondria. In conclusion, while Hibiscus appears primed for efficient photosynthesis and energy storage, Pelargonium may prioritise alternative cellular functions, engaging in a metabolic trade-off.

## 1. Introduction

The conversion of light into chemical energy by plants is fundamental to the global carbon cycle and sustains life on Earth [1]. The regulation of photosynthesis is a complex process, influenced by various structural, anatomical, and ultrastructural characteristics of plant tissues, particularly in leaves and chloroplasts, which impact both light and carbon fixation reactions [2]. Variations in these features often account for differences in photosynthetic rates, adaptability to environmental stresses, and ecological niches occupied by different plant species [1,3].

Photosynthetic efficiency is primarily driven by light absorption, and subsequent photochemical processes [1,4]. Upon light absorption by chloroplast pigments in light-harvesting complexes (LHCs), energy is transferred to reaction centres, initiating linear electron flow (LEF) [5,6] and converting light energy into chemical energy (primarily ATP (adenosine triphosphate) and NADPH (nicotinamide adenine dinucleotide phosphate)) [7,8]. A key aspect of this process is quantum yield, which defines the efficiency of photochemical energy transformation from light absorption to carbon fixation [9,10]. Other factors, such as stomatal conductance (*g*_s_) and intercellular CO_2_ concentration (*C*_i_), also play pivotal roles in modulating carboxylative processes [11,12]. Intrinsic water use efficiency (*i*WUE) is a crucial parameter for assessing a plant’s capacity to balance water consumption with carbon assimilation [13], demonstrating the intricate relationship between water use and photosynthesis. For example, an increase in *C*_i_ often correlates with an increase in photosynthetic rate [4,14], though it may also influence mesophyll conductance (*g*_m_) [12]. Conversely, reduced *g*_s_ can limit water loss through transpiration. High *i*WUE reflects a plant’s ability to efficiently manage water use while maintaining carbon absorption [1,15]. Understanding these interrelationships is crucial for advancing our understanding of photosynthetic regulation and its potential for optimisation.

Environmental factors such as temperature, light quality, and nutrient availability also significantly influence photosynthetic efficiency, underscoring the need to integrate these variables when optimising plant growth conditions [16,17]. Two critical parameters are the light compensation point (LCP) and the light saturation point (LSP), which contribute to variations in photosynthetic rates. The LCP denotes the light intensity where net photosynthesis is zero, balancing carbon gain from photosynthesis with losses from respiration and photorespiration. In contrast, the LSP denotes the point beyond which increases in light intensity no longer enhance photosynthesis. Advanced fluorescence analysis methods are used to evaluate photosynthetic performance and energy fluxes within photosystem II [18,19].

Carboxylation (*A*-*C*_i_) curve responses, which describe the relationship between photosynthetic rate and intercellular CO_2_ concentration, offer insights into a plant’s carbon fixation capacity [19,20]. Parameters such as *VC*_MAX_ (maximum carboxylation rate by RuBisCO), *AJ*_MAX_ (peak electron transport rate), and TPU (triose phosphate utilisation) define the metabolic limits of the photosynthetic apparatus [21]. The mesophyll conductance (gm) bridges internal CO_2_ diffusion with carboxylation processes [22]. For instance, high *VC*_MAX_ suggests robust carbon fixation, while elevated *AJ*_MAX_ indicates efficient electron transport during photosynthesis [22]. High TPU signifies the proficient utilisation of triose phosphates, and substantial gm reflects effective CO_2_ diffusion from intercellular spaces to chloroplasts [19,23].

Chlorophyll fluorescence, often overlooked, provides invaluable insights into photosynthetic function [21,24]. Parameters such as NPQ (nonphotochemical quenching), ΦPSII (quantum yield of photosystem II), ΦCO_2_ (photosystem II efficiency under elevated CO_2_), and ETR (electron transport rate) shed light on the dynamics of photosynthetic machinery [25,26]. Fv’/Fm’ (maximum quantum efficiency of PSII under light) and qP (photochemical quenching) serve as indicators of plant physiological health, offering benchmarks for comparing other results [25,26]. The JIP test further enhances our understanding of these metrics, revealing detailed insights into the functionality of photosystem II [27,28].

In photosynthetic research, the JIP test has become a good tool for analysing chlorophyll fluorescence and electron transport [27,28]. It allows for rapid and detailed analysis of photosystem II activity, with parameters like ϕ(PO), ϕ(EO), and PI(abs) offering critical insights into photosynthetic efficiency [29]. The strong correlation of these parameters with traditional photosynthetic metrics underscores their significance [30].

While the importance of chlorophyll fluorescence is widely acknowledged, there is limited understanding of how these parameters relate to the anatomical and ultrastructural features of plants [31]. Leaf morphology, such as thickness and stomatal distribution, and chloroplast ultrastructure, including thylakoid arrangement and plastoglobule presence, are likely linked to photosynthetic efficiency [22]. Moreover, cellular components like pigments and antioxidants can influence CO_2_ diffusion and the Calvin–Benson cycle. Spectral signatures of these compounds reveal critical insights into a plant’s physiological and metabolic status. Differences in structural and ultrastructural traits can result in significant variations in photosynthetic performance [21,32,33].

The genera Hibiscus and Pelargonium present an intriguing case for examining these relationships. Both are diverse and adapted to various ecological conditions, yet they exhibit distinct morphological and physiological traits. Hibiscus is characterised by robust grana structures and densely packed chloroplasts, which contribute to higher photosynthetic efficiency. In contrast, Pelargonium species possess fewer thylakoids and less dense chloroplasts, corresponding to lower photosynthetic performance [34,35,36]. These contrasting features provide a unique opportunity to explore the relationship between structure and function in photosynthesis and to assess their ecological implications. Furthermore, the role of mitochondria and other cytoplasmic components in energy metabolism has been less thoroughly explored, offering further avenues for investigation [37].

Therefore, this study aims to comprehensively compare the photosynthetic performance, leaf anatomy, and chloroplast ultrastructure of Hibiscus and Pelargonium. We hypothesise that Hibiscus species will exhibit higher photosynthetic efficiency and anatomical features optimised for energy conversion and storage, while Pelargonium will display structural and functional traits suggesting a metabolic trade-off, prioritising other cellular functions over optimal photosynthesis.

## 2. Results

### 2.1. Morphological Characteristics

**Figure 1:**
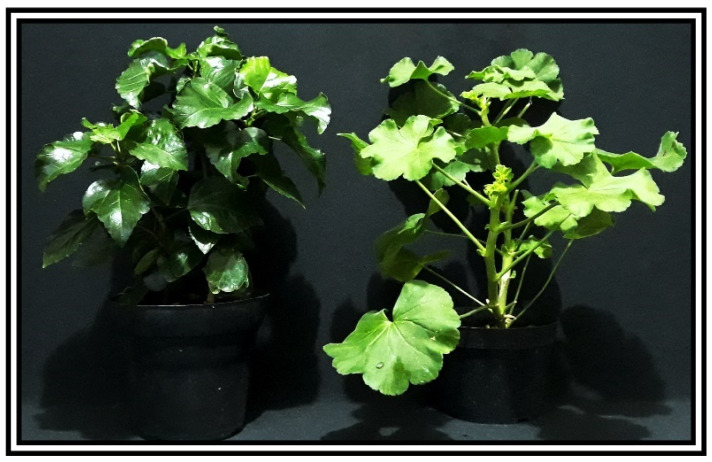
Representative of Hibiscus (*Hibiscus rosa-sinensis* L.) and Pelargonium (*Pelargonium zonale* (L.) L’Hér. Ex Aiton) plants. Hibiscus leaves exhibit a waxy surface and large size, while Pelargonium leaves are smaller, lobed, and covered with trichomes.

Representative Hibiscus (*Hibiscus rosa-sinensis* L.) and Pelargonium (*Pelargonium zonale* (L.) L’Hér. Ex Aiton) plants are shown in Figure 1. Hibiscus leaves predominantly exhibit a heart-shaped or ovate morphology and are often large enough to fit in the infrared gas analyser (IRGA) chamber without adjustments to leaf area, minimising measurement errors in IRGA assessments. The large leaf area potentially contributes to increased carbon fixation. Hibiscus leaves are characterised by a waxy epidermal layer, which likely aids in water retention, and exhibit an intense green shade (Figure 1, left). In contrast, Pelargonium leaves are smaller, with intricate lobing or serration, a lighter green colour, and a dense coverage of visible trichomes (Figure 1, right). The higher reflectivity indices in Pelargonium leaves, compared to Hibiscus, are visually apparent in Figure 1.

### 2.2. Leaf Optical Profile

**Figure 2:**
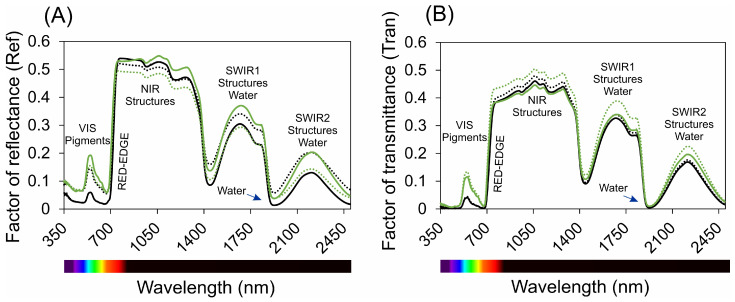

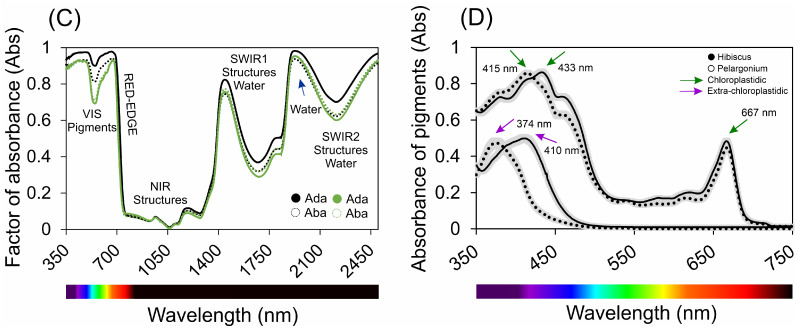
Spectral analysis of leaves (in vivo) and pigments (in vitro) in Hibiscus and Pelargonium plants. (**A**) Reflectance factor (Ref) from 350 to 2500 nm. (**B**) Transmittance factor (Trans) from 350 to 2500 nm. (**C**) Absorbance factor (Abs) from 350 to 2500 nm. (**D**) Spectral analysis of chloroplast and extrachloroplast pigments from 350 to 750 nm, with specific peaks for chlorophylls (green arrow) and flavonoids (pink arrow). The solid lines represent the adaxial surface, and the dashed lines represent the abaxial surface. The arrows highlight peaks for chlorophyll and flavonoid concentrations. Blue arrows denote water-specific spectral signatures. Peak shifts indicate variations due to pigments such as chlorophylls, carotenoids, and phenolic compounds. (*n* = 100).

The hyperspectral analysis of the leaves revealed distinct optical properties across various wavelength ranges, as shown in Figure 2. Both adaxial and abaxial surfaces were analysed for reflectance, transmittance, and absorbance factors. In the ultraviolet (UV) range (350–400 nm), both surfaces exhibited high absorbance and low reflectance. However, in the violet/blue range (400–450 nm), adaxial surfaces displayed lower reflectance and higher absorbance compared to abaxial surfaces, suggesting more efficient light absorption on the adaxial side.

In the blue/cyan range (450–495 nm), high absorbance values were noted for both surfaces, indicative of effective absorption of blue light by photosynthetic pigments within the leaf mesophyll. In the green range (495–570 nm), there was a marked increase in reflectance and transmittance, accompanied by a decrease in absorbance, with a notable minimum at 550 nm. Despite this, more than 70% of the green light was absorbed by both species, albeit less efficiently than other wavelengths.

A significant shift was observed in the red range (620–700 nm), where both surfaces exhibited increased absorbance and decreased reflectance, reflecting the high efficiency of red light in photosynthesis. Chlorophylls, rather than carotenoids, were identified as the primary pigments responsible for the high absorbance in this range. In the far-red and near-infrared (NIR) regions (700–1000 nm), both surfaces exhibited low absorbance and high reflectance, with light interacting in a complex manner beyond the visible spectrum. In the shortwave infrared (SWIR) spectrum (1300–2500 nm), absorbance levels increased significantly in the SWIR2 band (1800–2500 nm), compared to the SWIR1 band (1300–1800 nm).

Additionally, spectral analysis of extracted pigments revealed a peak flavonoid concentration at 410 nm in Hibiscus and 374 nm in Pelargonium (Figure 2D). These differences indicate the presence of distinct flavonoid compounds in each species. The mean flavonoid concentration was higher in Hibiscus (0.114 g m^−2^) than in Pelargonium (0.076 g m^−2^). Chloroplast pigments exhibited a peak at 433 nm in Hibiscus and 415 nm in Pelargonium, reflecting a variation in chlorophyll *a*/*b* ratios and carotenoid content (Figure 2D). Hibiscus had a slightly higher mean concentration of chloroplast pigments (*p* < 0.01) than Pelargonium, as indicated by the pink arrow in Figure 2D.

### 2.3. Pigments and Structural Components

**Figure 3:**
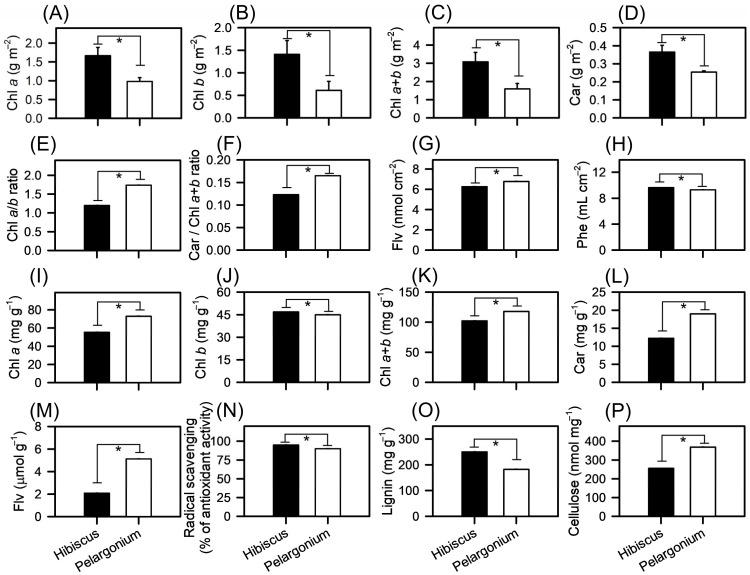
Concentrations of compounds in Hibiscus and Pelargonium plants. (**A**) Chlorophyll a (g m^−2^). (**B**) Chlorophyll b (g m^−2^). (**C**) Total chlorophyll (*a*+*b*) (g m^−2^). (**D**) Carotenoids (g m^−2^). (**E**) Chl a/b ratio. (**F**) Car/Chl a+b ratio. (**G**) Flavonoids (nmol cm^−2^). (**H**) Phenolic compounds (mL cm^−2^). (**I**) Chlorophyll a (mg g^−1^). (**J**) Chlorophyll b (mg g^−1^). (**K**) Total chlorophyll (a+b) (mg g^−1^). (**L**) Carotenoids (mg g^−1^). (**M**) Flavonoids (μmol g^−1^). (**N**) Radical scavenging (% of antioxidant activity). (**O**) Lignin (mg g^−1^). (**P**) Cellulose (nmol mg^−1^). Asterisks over bars indicate statistically significant differences in the *t*-test (*p* < 0.01). Mean ± SE (*n* = 100).

In our comprehensive physiological evaluation, several differences were observed across a range of physiological and biochemical markers (Figure 3). Starting with the chlorophyll concentration, Hibiscus exhibited a significantly higher amount of chlorophyll *a* at 1.67 g m^−2^, a value 69.9% greater than the 0.98 g m^−2^ observed in Pelargonium (*p* < 0.001) (Figure 3A). This trend was also consistent with the chlorophyll b concentrations: Hibiscus had a concentration of 1.41 g m^−2^, which is 130.9% higher than Pelargonium’s 0.62 g m^−2^ (*p* < 0.001) (Figure 3B). Extending this comparison to total chlorophyll (*a*+*b*) concentrations (Figure 3C–G), the total for Hibiscus was 3.1 g m^−2^, a 93.5% increase relative to Pelargonium’s 1.6 g m^−2^. This difference was statistically significant (*p* < 0.001) (Figure 3A–L).

Turning to other compounds, the concentration of flavonoids was higher in Pelargonium, with a 144.7% increase, reaching 5.14 μmol g^−1^ compared to 2.10 μmol g^−1^ in Hibiscus. This difference was statistically significant, with a *p*-value of less than 0.001 (Figure 3G,M). Hibiscus showed a subtle yet significant 3.6% increase in the concentration of phenolic compounds, reaching 9.63 mL cm^−2^, as opposed to Pelargonium’s 9.30 mL cm^−2^ (Figure 3H). Additionally, Hibiscus demonstrated a 5.5% increase in DPPH reagent concentration, reaching 95.02 compared to Pelargonium’s 90.09, a statistically significant difference (*p* < 0.001) (Figure 3N).

Regarding structural components, Hibiscus showed a considerable 37.0% increase in lignin content (250.5 mg g^−1^), which was notably higher than Pelargonium’s 182.8 mg g^−1^ (Figure 3O). Nonetheless, this was expected for a woody plant compared to an herbaceous plant. In contrast, Pelargonium had a higher cellulose concentration, with a 44.1% increase, reaching 369.3 nmol mg^−1^ MS compared to Hibiscus’s 256.1 nmol mg^−1^ MS (Figure 3P).

### 2.4. Diurnal Gas Exchange

**Figure 4:**
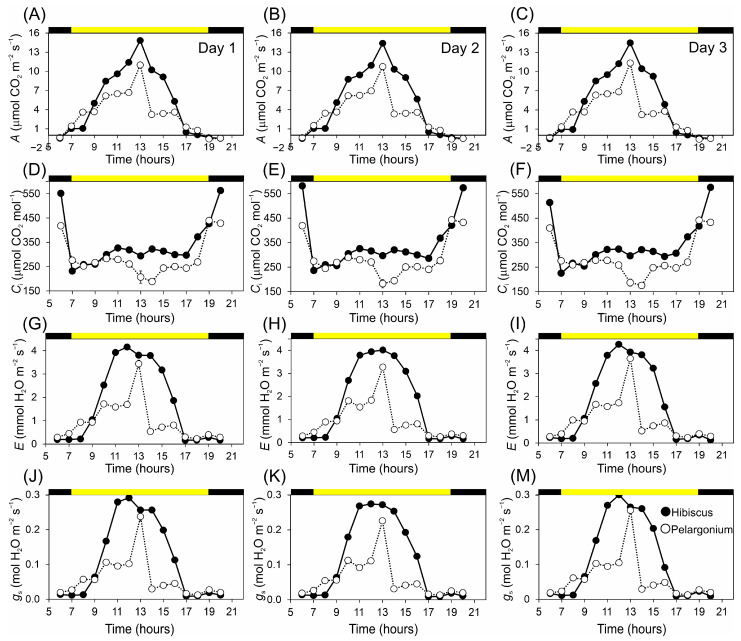
Daily curves between 6 and 20 h were evaluated over three days for Hibiscus and Pelargonium plants. (**A**–**C**) Net assimilation rate (μmol CO_2_ m^−2^ s^−1^). (**D**–**F**) Internal CO_2_ concentration (μmol CO_2_ mol^−1^). (**G**–**H**) Net transpiration rate (mmol H_2_O m^−2^ s^−1^). (**J**–**M**) Stomatal conductance (mol H_2_O m^−2^ s^−1^). Black bars indicate darkness, and yellow bars indicate light environments. Mean ± SE (*n* = 20).

Over a three-day period, we observed diurnal fluctuations in the net photosynthetic rate in Hibiscus and Pelargonium, recording four key physiological parameters: net carbon assimilation rate (*A*), internal CO_2_ concentration (*C*_i_), net transpiration rate (*E*), and stomatal conductance (*g*_s_) (Figure 4). Hibiscus consistently showed higher *A* across the three days of analysis, reaching a maximum of 14.56 µmol CO_2_ m^−2^ s^−1^ at 1 p.m. and a minimum of −0.52 µmol µmol CO_2_ m^−2^ s^−1^ at 6 a.m., during the dark period when measurements recorded dark respiration (Rd). In contrast, Pelargonium reached a peak of 11.01 µmol µmol CO_2_ m^−2^ s^−1^ at 1 p.m. and a lower value of −0.60 µmol µmol CO_2_ m^−2^ s^−1^ recorded at 7 p.m. (Figure 4A–C).

In addition, Hibiscus also presented a higher *C*_i_ throughout the entire period, reaching 571 µmol mol^−^¹ at 8 p.m. and a low of 231 µmol mol^−1^ at 7 a.m. We highlight that when *C*_i_ values are larger than *C*_a_ (ambient CO_2_ concentration, typically 400 µmol mol^−1^), the net carbon exchange shows negative values. Pelargonium exhibited a maximum concentration of 441 µmol mol^−1^ at 7 p.m. and a minimum concentration of 186 µmol mol^−1^ at 2 p.m. (Figure 4D–F).

In general, the *E* values were consistent with the *g*_s_ values. *E* in Hibiscus was higher than in Pelargonium, peaking at 4.12 mmol H_2_O m^−2^ s^−1^ at midday, with a low value of 0.15 mmol H_2_O m^−2^ s^−1^ at 8 p.m. For Pelargonium, the highest rate was 3.45 mmol H_2_O m^−2^ s^−1^ at 1 p.m., and the lowest was 0.24 mmol H_2_O m^−2^ s^−1^ at 6 p.m. (Figure 4G–I). Additionally, Hibiscus showed higher *g*_s_ (0.29 mol H_2_O m^−2^ s^−1^) at midday and lower gs (0.009 mol H_2_O m^−2^ s^−1^) at sunset, while Pelargonium exhibited a peak of 0.24 mol H_2_O m^−2^ s^−1^ at 1 p.m. and a minimum of 0.013 mol H_2_O m^−2^ s^−1^ at 6 p.m. (Figure 4J–M).

### 2.5. Photosynthetic Response to Light and CO_2_

**Figure 5:**
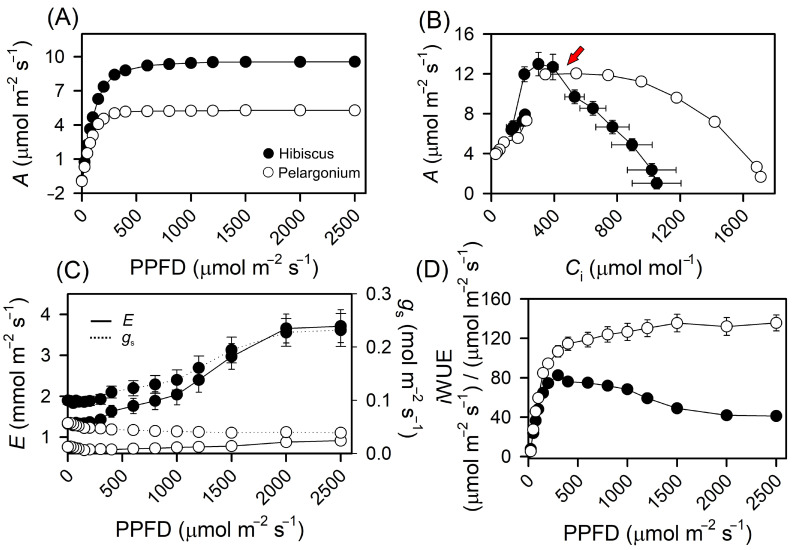
Response curves for Hibiscus and Pelargonium plants. (**A**) Net photosynthetic light (*A*-PPFD) response. (**B**) Net photosynthetic CO_2_ (*A*−*C*_i_) responses. (**C**) Stomatal conductance (*g*_s_) and transpiration rate (*E*). (**D**) Intrinsic water use efficiency (*i*WUE) response curves. The red arrow indicates the inflection point of 426 μmol mol^−1^ CO_2_ for decreased *C*_i_ in leaves. Mean ± SE (*n* = 10).

At the lowest PPFD level, Hibiscus exhibited a net photosynthesis rate (*A*) approximately 5.3% lower than that of Pelargonium (Figure 5). Conversely, at the highest PPFD level, Hibiscus demonstrated an 81% increase in *A* compared to Pelargonium (Figure 5A and Table 1). A similar trend was observed for *C*_i_ (Figure 5B), where Hibiscus presented *C*_i_ values 5.1% lower than Pelargonium at the lowest PPFD level but 20.6% higher at the highest PPFD level (Figure 5A,B and Table 1).

As expected from daily photosynthetic analysis, Hibiscus had higher *g*_s_ than Pelargonium at both the lowest and highest PPFD values, exceeding Pelargonium by 74.6% and 495.9%, respectively (Figure 5C). E exhibited a parallel pattern, with a 75.2% increase in Hibiscus at the lowest PPFD level and a 306.3% increase at the highest PPFD level (Figure 5C). These data indicate that, although Hibiscus initially displayed lower *A* and *C*_i_ values than Pelargonium, it significantly improved these metrics under higher PPFD levels. Hibiscus consistently maintained higher *E* and *g*_s_ values at both low and high PPFD (Figure 5C,D).

The intrinsic water-use efficiency (*i*WUE) was 40.13% higher in Hibiscus at the lowest PPFD level, but 69.63% lower at the highest PPFD level compared to Pelargonium (Figure 5D; inset). This “opposite” behaviour is likely related to the higher *g*_s_ and *E* observed in Hibiscus plants, despite the increase in *A*, *E*, and *g*_s_ at higher rates.

### 2.6. Fluorescence Measurementsin Leaves

**Figure 6:**
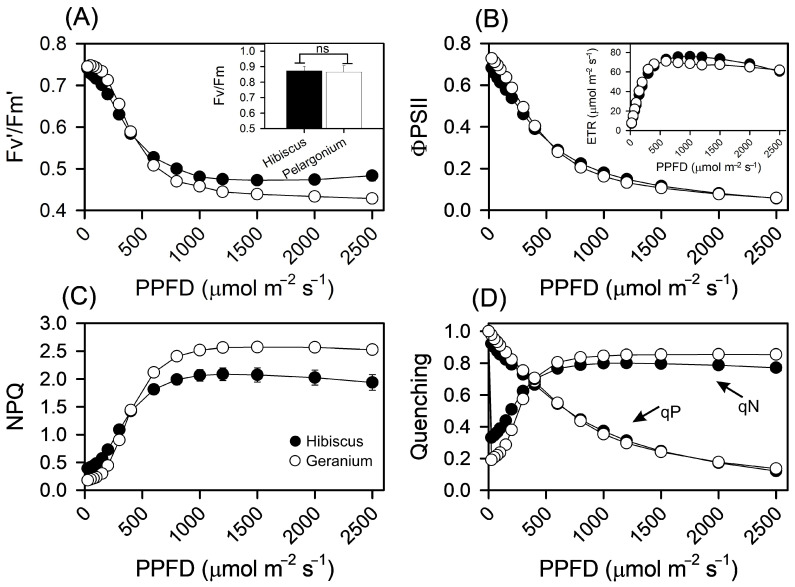
Fluorescence response curves obtained simultaneously with the photosynthetic response to light in Hibiscus and Pelargonium plants. (**A**) Effective quantum yield of PSII (Fv’/Fm’). The inset shown in the bar graph indicates the maximum quantum yield of PSII (Fv/Fm) in dark−adapted leaves. (**B**) Operational efficiency of photosystem II (ΦPSII). The inset shows the electron transport rate (ETR). (**C**) Nonphotochemical quenching (NPQ). (**D**) Photochemical dissipation quenching (qP) and nonphotochemical dissipation quenching (qN). Asterisks over the bars indicate statistically significant differences according to the t-test (*p* < 0.01). “ns” denotes no statistical significance. Mean ± SE (*n* = 10).

The maximum quantum yield of PSII in dark-acclimated plants (Fv/Fm) was similar in Hibiscus and Pelargonium. Under non-stressed conditions, plants typically exhibit values around 0.82 (±0.02), which serves as a good initial predictor of photochemical efficiency. Under stressed conditions, lower values (<0.60), along with light-acclimated parameters such as Fv’/Fm’ (PSII operating efficiency), provide a better estimation of the efficiency with which light absorbed by PSII is used for quinone A (Q_A_) reduction by Baker et al. [25]. The photochemical efficiency of PSII (ΦPSII), which reflects the use of excitation energy within PSII to drive electron transport from P680 to Q_A_, was 6.18% lower in Hibiscus at the lowest PPFD level and 2.30% lower at the highest PPFD level compared to Pelargonium (Figure 6B). Furthermore, the electron transport rate (ETR) followed similar trends, with Hibiscus showing an ETR that was 6.26% lower at the lowest PPFD and 2.30% lower at the highest PPFD level than Pelargonium (Figure 6B; inset).

Non-photochemical quenching (NPQ), which is associated with light absorption not coupled with electron loss at P680 and involves heat dissipation from PSII, was particularly pronounced in Hibiscus. At the lowest PPFD, Hibiscus exhibited an NPQ value 119.41% higher than Pelargonium, and 23.24% higher at the highest PPFD level (Figure 6C). Additionally, the photochemical coefficient (qP) in Hibiscus was 5.66% and 10.94% lower than in Pelargonium at the lowest and highest PPFD levels, respectively (Figure 6D). Regarding other non-photochemical quenching estimators, such as qN, Hibiscus exhibited a value 73.22% higher at the lowest PPFD but 9.70% lower at the highest PPFD compared to Pelargonium (Figure 6D).

### 2.7. Chlorophyll a Fluorescence Kinetic Dynamics

**Figure 7:**
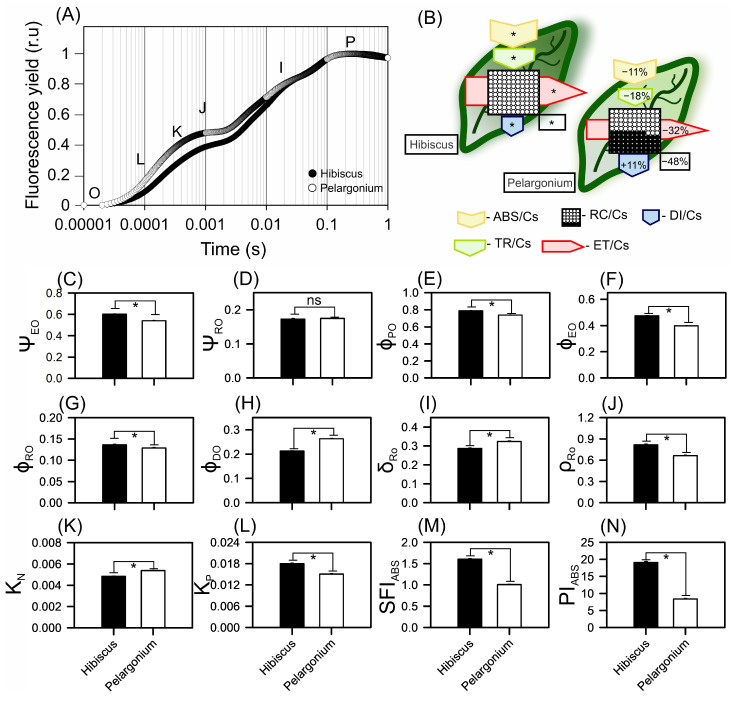
Chlorophyll a fluorescence kinetic parameters derived from the JIP test in Hibiscus and Pelargonium plants. (**A**) Chlorophyll a fluorescence induction kinetics using normalised data. (**B**) Pipeline leaves display phenomenological energy flow through the excited cross-sections (CSs) of leaves. Yellow arrow—ABS/CS, absorption flow by approximate CS; green arrow—TR/CS, energy flow trapped by CS; red arrow—ET/CS, electron transport flow by CS; blue arrow—DI/CS, energy flow dissipated by CS; circles inscribed in squares—RC/CS indicate the % of active/inactive reaction centres. The white circles inscribed in squares represent reduced (active) QA reaction centres, the black circles represent non-reducing (inactive) QA reaction centres, and 100% of the active reaction centres responded with the highest average numbers observed in relation to Hibiscus. Arrow sizes indicate changes in the energy flow to Hibiscus plants. (**C**) ΨEO. (**D**) ΨRO. (**E**) ΦPO. (**F**) ΦPO. (**G**) ΦRO. (**H**) ΦDO. (**I**) δRO. (**J**) ρRO. (**K**) KN. (**L**) KP. (**M**) SFI_ABS_. (**N**) PI_ABS_. Different asterisks inside the arrows indicate significance, as determined by a *t*-test (*p* < 0.01). Mean ± SE (*n* = 100).

Based on the JIP test, chlorophyll a fluorescence kinetics indicated that the parameters ϕ(PO), ϕ(EO), and PI(abs) increased in Hibiscus by 6.85% (Figure 7E), 18.91% (Figure 7F), and 127.63% (Figure 7N), respectively. In contrast, variables such as ϕ(DO) and δRo showed decreases in Hibiscus of 19.17% (Figure 7H) and 11.34% (Figure 7I), respectively. The variable ρRo (Figure 7J) increased by 22.73% in Hibiscus, whereas Kn and ABS/RC decreased by 10.03% (Figure 7K) and 25.20% (Figure 7B), respectively.

The phenomenological fluxes by the RC/CS ratio, which reflects the density of reaction centres per unit of chlorophyll, increased by 48.35% in Hibiscus compared to Pelargonium plants (Figure 7B). Similarly, ABS/CS, which represents the absorption of light energy per unit leaf cross-section, increased by 10.91% in Hibiscus. The TRo/CS parameter, an indicator of energy-trapping efficiency, was elevated by 18.49% in Hibiscus relative to Pelargonium. The ETo/CS, which measures the electron transport rate, also increased by 31.84% in Hibiscus compared to Pelargonium. In contrast, the DIo/CS parameter, which represents the energy dissipated per active reaction centre, decreased by 10.34% in Hibiscus compared to Pelargonium (Figure 7B). The reduced dissipation of absorbed light energy in Hibiscus was aligned with the fluorescence curves (Figure 7A). Additionally, Hibiscus exhibited higher mean values than Pelargonium for all phenomenological fluxes, with the exception of DIo/CS (Figure 7B).

### 2.8. Anatomy, Structure and Ultrastructure

**Figure 8:**
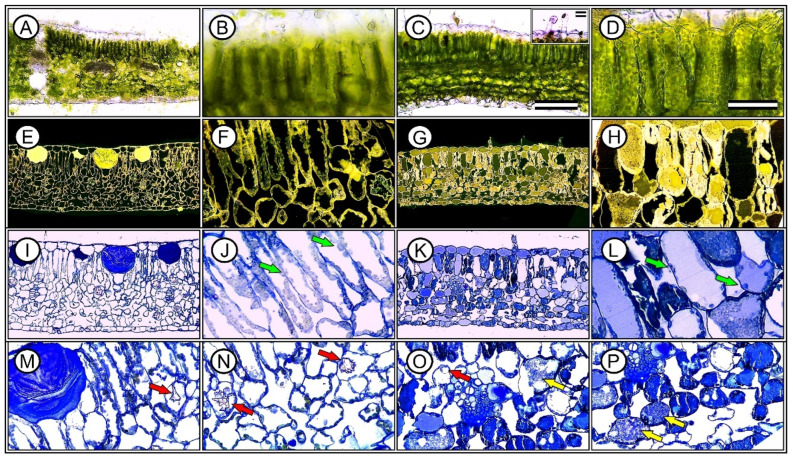
Representative images of optical microscopy (OM) in top–bottom and anatomical analyses of Hibiscus (first and second columns) and Pelargonium (third and fourth columns) plants. (**A**–**D**) Cross-sections. (**E**–**H**) Historesin cross-sections under false colour. (**I**–**L**) Details of the leaf thickness and cells. (**M**–**P**) Structures present in cellular tissues. Green arrows indicate chloroplasts, red arrows indicate diffuse crystals, and yellow arrows indicate dense cytoplasmic content. Accumulative and secretory structures of the adaxial epidermis are highlighted. Scale bars = 200 µm and 50 µm, left to right, respectively.

**Figure 9:**
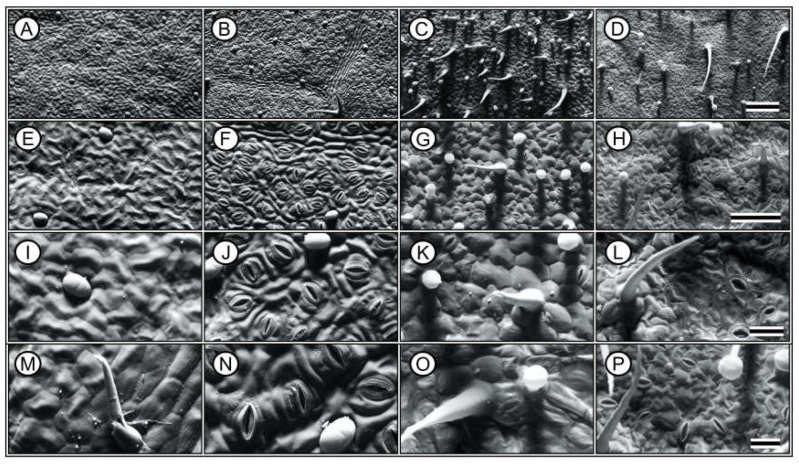
Representative scanning electron microscopy (SEM) images of adaxial and abaxial surfaces of Hibiscus and Pelargonium plants. (**A**,**E**,**I**,**M**) Adaxial surface of the Hibiscus. (**B**,**F**,**J**,**N**) Abaxial surface of the Hibiscus. (**C**,**G**,**K**,**O**) Adaxial surface of Pelargonium. (**D**,**H**,**L**,**P**) Abaxial surface of Pelargonium. Scale bars = 250 μm (**A**–**D**), 150 μm (**E**–**H**), and 50 μm (**I**–**P**), top to bottom, respectively.

**Figure 10:**
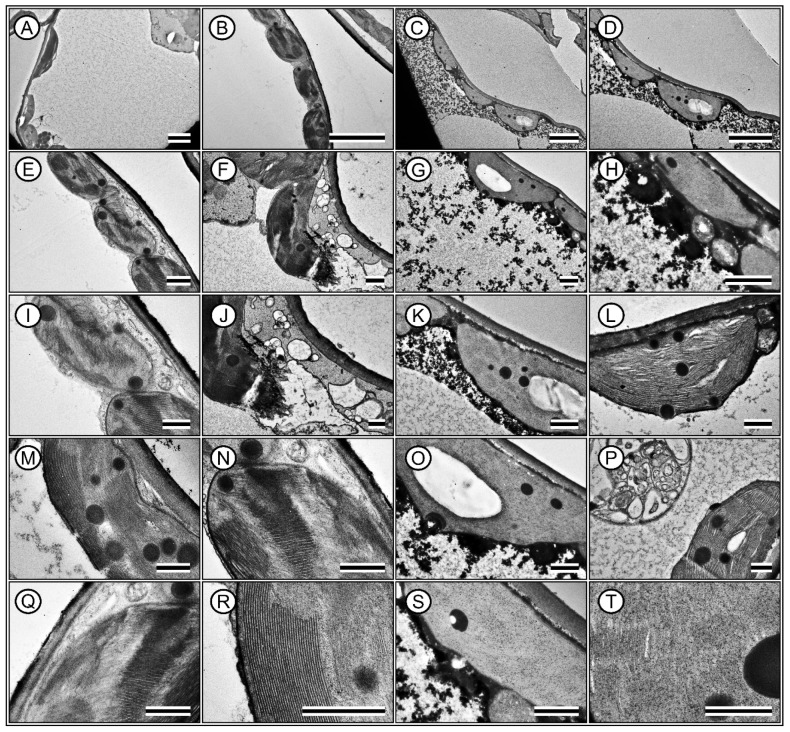
Representative transmission electron microscopy (TEM) images of chloroplasts in Hibiscus and Pelargonium plants. (**A**,**B**,**E**,**F**,**I**,**J**,**M**,**N**,**Q**,**R**) Hibiscus. (**C**,**D**,**G**,**H**,**K**,**L**,**O**,**P**,**S**,**T**) Pelargonium plants. Scale bar = 4 μm (**A**–**D**), 1 μm (**E**–**P**) and 600 nm (**Q**–**T**).

**Figure 11:**
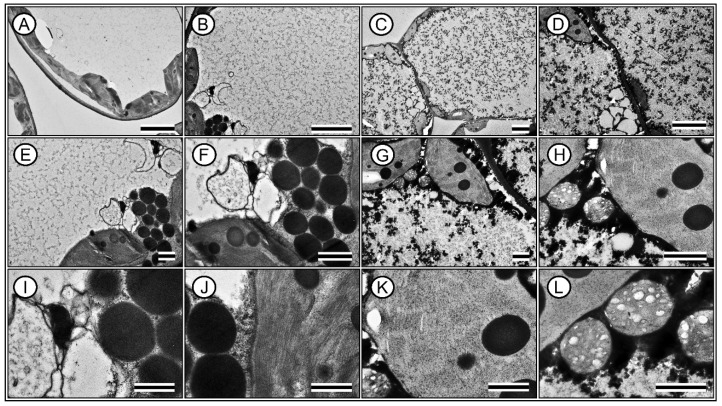
Representative transmission electron microscopy (TEM) images of mesophyll cells in the leaves. (**A**,**B**,**E**,**F**,**I**,**J**) Hibiscus. (**C**,**D**,**G**,**H**,**K**,**L**) Pelargonium plants. Scale bar = 4 μm (**A**–**D**), 1 μm (**E**–**P**) and 600 nm (**Q**–**T**).

A comparative analysis of the leaf parameters between Hibiscus and Pelargonium plants revealed several notable differences (Figure 8, Figure 9, Figure 10 and Figure 11). First, the characteristics of the adaxial and abaxial surfaces contributed to changes in optical properties, stomatal density and size, and the presence of trichomes (Figure 8 and Figure 9; Table 2). The leaf thickness (LFT) in Hibiscus was approximately 4.98% greater than in Pelargonium (Figure 8 and Table 2). Similarly, the thicknesses of the palisade parenchyma (PLT) and spongy parenchyma (SPT) were 16.62% and 6.49% thicker in Hibiscus, respectively. However, Hibiscus exhibited a thinner adaxial leaf epidermis (AdLE) and abaxial leaf epidermis (AbLE) by 9.62% and 21.45% (*p* < 0.001), respectively, compared to Pelargonium (Figure 8 and Table 2).

In terms of the spongy parenchyma-to-palisade parenchyma ratio (SPL ratio), Hibiscus displayed a value approximately 8.72% lower than Pelargonium (*p* < 0.001; Figure 8 and Figure 9; Table 2). Stomata on the adaxial surface (ASD) were absent in Hibiscus, which is common in woody plants but were present in Pelargonium. On the abaxial side, stomatal density (ABD) in Hibiscus was 98.06% greater than in Pelargonium (Figure 8). Adaxial trichomes (AdTT) were absent in Hibiscus but present in Pelargonium. Conversely, the abaxial trichome density (AbTT) was 69.86% lower in Hibiscus, while the density of glandular trichomes on both the adaxial (AdGT) and abaxial (AbGT) sides was 53.08% and 51.99% lower, respectively (Figure 9 and Table 2).

Hibiscus had a significantly higher chloroplast density than Pelargonium, with 50.55% more chloroplasts in the palisade parenchyma (PLC) and 84.21% more chloroplasts in the spongy parenchyma (SPC) (Figure 8 and Figure 10). Additionally, Hibiscus displayed a higher spongy-to-palisade chloroplast ratio (SPC ratio) by approximately 23.28% (Table 2). Qualitatively, Hibiscus exhibited a more robust organisation of grana with superdense stacking and a diminished stromal region relative to the lamellae. A high accumulation of plastoglobules was also observed (Figure 10 and Figure 11; Table 2).

The photochemical and carboxylative efficiencies of plants depend on the intensity and quality of light reaching the reaction centres, but they are more strongly associated with the ability of chloroplasts to absorb light. Some wavelengths may be more efficient due to their penetrability into deeper leaf layers or the distribution of chloroplasts, particularly in the spongy parenchyma, which maximises the formation of reducing power (ATP and NADPH).

In contrast, chloroplasts in Pelargonium were sparse (Figure 10 and Figure 11). The chloroplasts that were present showed typical structures but with fewer thylakoids, suggesting limited functionality (Figure 10). These chloroplasts also showed minimal evidence of plastoglobules (Figure 11). Although Pelargonium chloroplasts accumulated starch, the low electron density suggested limited functionality compared to Hibiscus (Figure 10 and Figure 11).

Hibiscus cells were rich in mucilage, while Pelargonium’s cytoplasm contained a high proportion of residual structures or unidentified substances despite having numerous mitochondria positioned adjacent to the chloroplasts, indicating close spatial association, predominantly displaying a globular or rounded shape. (Figure 10 and Figure 11).

### 2.9. Principal Component Analysis

**Figure 12:**
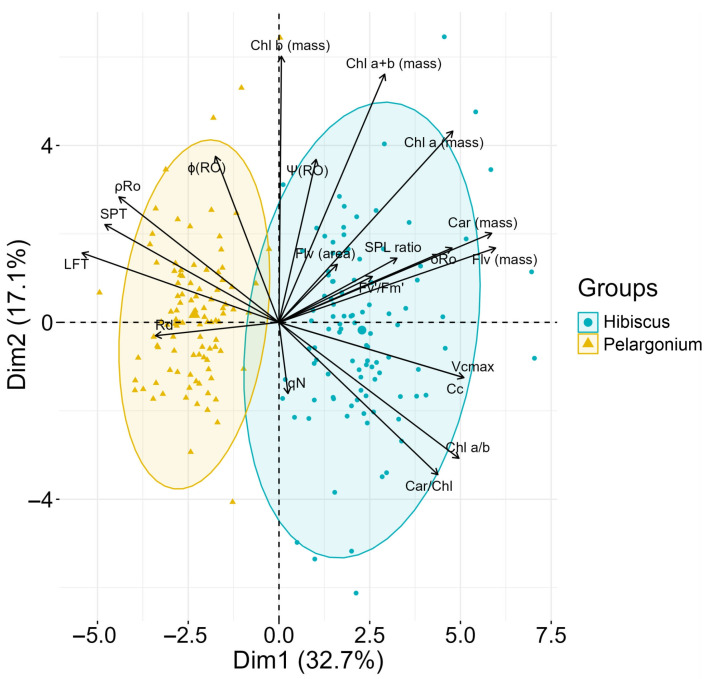
Multivariate analysis of Hibiscus and Pelargonium plants. The 2D PCA biplot of principal component analysis (PCA) displayed two dimensions (Dim1 and Dim2) and the contribution of the 20 most important variables to explain the formed clusters. See the abbreviation in Section 4.

Principal component analysis (PCA) was conducted to optimise the variance in a linear combination of variables by identifying dimensions along which observations are maximally separated based on their scores, providing a single scale with unequal weights to delineate treatments. This PCA aimed to investigate the relationships between the 20 most responsive variables (among the 74 variables analysed) that correlated with both Hibiscus and Pelargonium species. The first two principal components accounted for 49.8% of the total variance, with Dim1 and Dim2 explaining 32.7% and 17.1% of the variance, respectively (Figure 12).

**Figure 13:**
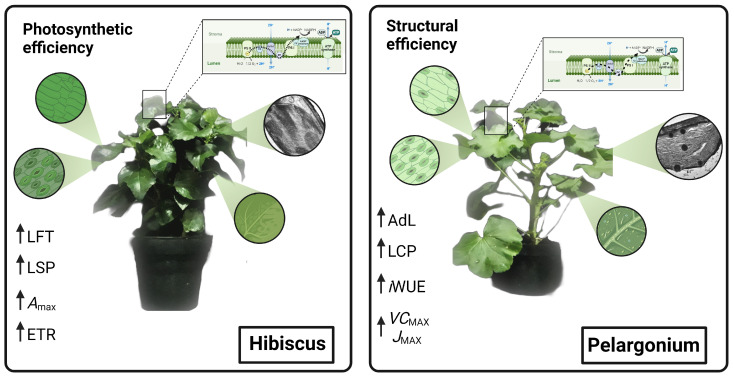
Comparative scheme of Hibiscus and Pelargonium plants. It highlights the superior photosynthetic efficiency of Hibiscus, emphasising its enhanced cellular structure, including higher chloroplast density, which contributes to improved photosynthesis and energy storage. In contrast, Pelargonium exhibits cellular adjustments, including changes in thylakoid count and a higher proportion of mitochondria, suggesting resource allocation to alternative cellular functions. Detailed insets and labels elucidate the distinct morphological, biochemical, and photosynthetic adaptations between the two species. Thicker lines indicate more efficient electron flow in the electron transport chain. Elements of the figure were created using Biorender.com (accessed on 5 October 2024).

Hibiscus plants were more strongly associated with pigment concentrations expressed per unit mass, as well as with parameters related to photochemical efficiency (Figure 13). The cluster formed for Hibiscus was more dispersed, but of the top 20 variables, 13 were strongly correlated with this species (Figure 12; light blue clustering). In contrast, Pelargonium plants showed a greater association with parameters related to non-photochemical efficiency, such as thermal dissipation and fluorescence, as well as variables related to leaf mesophyll structure. Five variables were most strongly correlated with Pelargonium plants, with more compact clustering (Figure 12; light orange clustering). These PCA findings suggest significant differences in the core physiological performance between the two species (Figure 12 and Figure 13).

## 3. Discussion

### 3.1. Hyperspectral Leaf Optical Proprieties

The data collected from the morphological, anatomical, structural, ultrastructural, and biochemical features, along with their optical properties in the leaves of Hibiscus and Pelargonium, expand our understanding of the photochemical and carboxylative properties of these species. Some differences can be attributed to the adaxial or abaxial surfaces, such as stomatal size, density, and the presence of trichomes, which contribute to their optical signatures. Other variations are linked to differences in the palisade parenchyma, chloroplast density, and photosynthetic pigments. These findings enhance our knowledge of the complex interactions between plants and their environments. An integrative analysis of Hibiscus and Pelargonium revealed differences in the reflective indices of their leaves, which were associated with changes in their photosynthetic capacity (Figure 1, Figure 2, Figure 3, Figure 4, Figure 5, Figure 6, Figure 7, Figure 8, Figure 9, Figure 10, Figure 11, Figure 12 and Figure 13).

Numerous studies have examined the adaptations of adaxial and abaxial leaf surfaces in relation to light absorption and photosynthetic processes [38,39,40]. The high absorptance indices in the ultraviolet (UV) range in both species can be attributed to the presence of photoprotective pigments, such as phenolic compounds, which play a crucial role in shielding against UV radiation and mitigating UV-induced damage, often associated with heat stress [41,42]. Additionally, variations in reflectance between the adaxial and abaxial surfaces, particularly in the violet and blue regions, can be linked to the general anatomical structure of the leaves. These variations may enhance photosynthetic capacity by improving the plant’s ability to use available light more efficiently, especially when light enters through the adaxial surface.

Morphological investigations suggest that the abaxial epidermal cells typically possess a higher density of trichomes and a more irregular structure, influencing their optical properties [41,43]. The fluctuation in absorptance within the green wavelength range between adaxial and abaxial regions could be influenced by cell density and chloroplast distribution [44]. The high blue and red absorptance, along with the lower green light absorptance, indicate that blue and red wavelengths are absorbed more superficially by the chlorophylls, while green light penetrates deeper into the spongy parenchyma due to its higher transmission [45].

The low absorptance in the near-infrared (NIR) and far-infrared (FIR) regions may stem from the absence of highly absorbing compounds, though these ranges significantly influence plant development through phytochrome-mediated processes, such as photomorphogenesis and the shade-avoidance response [46]. These spectral differences can impact photosynthetic efficiency and plant adaptation strategies across diverse light environments. In this context, hyperspectral analysis of adaxial and abaxial leaf surfaces offers valuable insights into their optical and adaptive properties [35]. Applying these findings to an eco-physiological and whole-plant context provides deeper insights into how plants optimise light absorption and adjust their photosynthetic strategies in response to varying environmental conditions. These adaptive strategies help plants succeed in a wide range of light-limited or stress-inducing environments [32,47,48,49].

### 3.2. Changes in Photosynthesis-Related Compound Levels

The comparative analysis of photosynthetic compounds in Hibiscus and Pelargonium revealed variations in chlorophyll, carotenoids, flavonoids, phenolic compounds, antioxidant capacity, and structural components such as lignin and cellulose (Figure 3). The elevated levels of chlorophyll in Hibiscus suggest a more efficient light-harvesting capability, as chlorophyll plays a pivotal role in photosynthesis and influences leaf optical properties (Figure 3, Figure 4, Figure 5 and Figure 6). These findings align with previous studies that highlight the positive correlation between chlorophyll content and photochemical efficiency [50,51,52]. In contrast, Pelargonium exhibited a significantly higher carotenoid-to-chlorophyll ratio. Since carotenoids function as antioxidants and protect against photooxidative damage, this suggests that Pelargonium may be better equipped to handle environmental stressors such as high light intensity or nutrient deficiency, corroborating earlier research [53,54].

Pelargonium also displayed particularly high flavonoid concentrations (Figure 3). Flavonoids are known for their role in plant defence mechanisms against herbivores and pathogens [48,55]. The elevated concentrations in Pelargonium could provide a biological advantage in coping with biotic stress. On the other hand, Hibiscus showed a marginally higher concentration of phenolic compounds, which are typically associated with resistance to abiotic stresses, such as UV radiation. The observed shift in absorption peaks from 374 nm (0.47) in Pelargonium to 410 nm (0.49) in Hibiscus suggests the presence of distinct phenolic compounds in the two species. Additionally, the DPPH reagent concentration was slightly higher in Hibiscus, indicating superior total antioxidant capacity, as antioxidants are crucial in mitigating oxidative stress [56,57].

Hibiscus exhibited higher lignin content (Figure 3), a structural polymer that strengthens cell walls and is associated with resistance to pathogens and mechanical stress [52,58,59]. This is consistent with its classification as a woody plant, although the lignin was measured in the leaves, not the stems. In contrast, Pelargonium had significantly higher cellulose content, which may confer greater structural stability or faster growth rates, as cellulose is the primary structural component of plant cell walls. These structural components may also affect CO_2_ diffusion within the leaf, as CO_2_ must navigate the mesophyll to reach chloroplasts. Recent studies suggest that lignification can hinder CO_2_ diffusion [60].

Hibiscus displayed a pronounced reduction in its *A*-*C*_i_ curves compared to Pelargonium, likely due to increased lignification, which impedes CO_2_ diffusion (Figure 5B). A reduced lignin concentration, coupled with higher cellulose levels in Pelargonium, could enhance CO_2_ accessibility to chloroplasts by minimising barriers such as thickened cell walls. The inflection point of 426 μmol mol^−1^, observed between the two species, highlights the importance of biochemical adjustments in optimising photosynthesis under different CO_2_ concentrations (Figure 5A,B) [54,61,62,63].

The distinct biochemical and structural profiles of Hibiscus and Pelargonium likely provide these species with unique ecological niches, survival strategies, and interactions with their environments. These findings have significant implications for agricultural practises, particularly in selecting species for specific ecological roles or stress conditions [35,54,61,62,63,64].

### 3.3. Diurnal Photosynthesis

The diurnal photosynthetic activity dataset collected over three days minimised the impact of uncontrollable variables inherent in single-day analyses (Figure 4). The data revealed significant differences between Hibiscus and Pelargonium, shedding light on their possible physiological variations and ecological roles [65,66]. For instance, in terms of carbon fixation, Hibiscus consistently exhibited higher net carbon assimilation rates than Pelargonium and other ornamental plants (Figure 5 and Table 1). The peak assimilation rate of 14.56 µmol CO_2_ m^−2^ s^−1^ in Hibiscus was notably higher than the peak rate of 11.01 µmol CO_2_ m^−2^ s^−1^ recorded in Pelargonium. This marked difference suggests that Hibiscus possesses an enhanced carbon fixation capacity, potentially providing it with an ecological advantage in environments where carbon resources are abundant. Such a high carbon assimilation rate hints at a more efficient Calvin cycle, likely allowing Hibiscus to demonstrate more vigorous growth and competitive ability in relation to other species [67,68]. Moreover, Hibiscus displayed consistently higher levels of internal CO_2_ concentration (*C*_i_) throughout the day, which was correlated with its higher stomatal conductance (*g*_s_). This elevated *C*_i_ supports a higher rate of net assimilation (*A*), assuming mesophyll conductance is not a limiting factor. However, this also resulted in a higher transpiration rate (*E*), suggesting increased water usage (Figure 5).

The transpiration rate in Hibiscus peaked at 4.12 mmol µmol H_2_O m^−2^ s^−1^, compared to 3.45 mmol H_2_O m^−2^ s^−1^ in Pelargonium. This difference could be both beneficial and detrimental: on one hand, increased transpiration might facilitate greater nutrient uptake, thereby enhancing overall fitness in Hibiscus. On the other hand, this trait could also make Hibiscus more susceptible to water stress, particularly in arid environments or under drought conditions [22,34]. In this context, stomatal conductance plays a critical role in regulating CO_2_ uptake and water loss in both species [22,34]. The balance between carbon assimilation and water loss, as reflected in these findings, underscores the importance of water-use efficiency (WUE) in determining the ecological fitness of these plants.

Consequently, the observed increase in photosynthetic activity presents important ecological trade-offs for both carbon allocation to cellular structures and water-use efficiency [13,69]. These findings suggest that Hibiscus and Pelargonium have evolved distinct adaptive strategies, which may ultimately influence their resilience and distribution across various ecosystems, particularly those limited by resources such as water and nutrients. Hibiscus, with its higher growth rates and photosynthetic performance, may thrive in environments where resources are more abundant, while Pelargonium may be better suited to more resource-limited conditions.

### 3.4. Photosynthetic Analyses

The distinct responses of net assimilation rate (*A*) to varying light and CO_2_ concentrations in Hibiscus and Pelargonium (Figure 5 and Table 1) provide key insights into how different species adjust their photosynthetic machinery. At lower CO_2_ concentrations, Hibiscus exhibited a significantly reduced net carbon fixation rate and lower internal CO_2_ concentration (*C*_i_) compared to Pelargonium. This may be due to Hibiscus’s higher capacity for internal CO_2_ fixation without a corresponding increase in CO_2_ influx through the stomata, leading to a feedforward mechanism that reduces photosynthetic efficiency under low CO_2_ conditions. Lower *C*_i_ could further reduce chloroplast CO_2_ concentration (*C*c), limiting RuBisCO’s efficiency [70,71]. However, Hibiscus overcame this limitation at higher CO_2_ concentrations, showing an 8.1% increase in *A* compared to Pelargonium (Figure 5A,B; red arrow), indicating an adaptive mechanism that enhances photosynthetic efficiency under elevated CO_2_ conditions [72]. This trend aligns with Hibiscus’s higher daily stomatal conductance (*g*_s_) (Table 1 and Figure 4 and Figure 5) [18].

Despite this, at the highest CO_2_ levels in the infrared gas analyser (IRGA) chamber (2000 µmol mol^−1^), Hibiscus still exhibited lower *C*_i_ values than Pelargonium (Figure 4 and Figure 5). This suggests potential limitations in Hibiscus’s ability to regulate internal CO_2_ concentrations, even under elevated atmospheric CO_2_. Further research is needed to determine whether these differences in carbon fixation and internal CO_2_ concentration between Hibiscus and Pelargonium are indicative of adaptive strategies or physiological constraints [73].

In conclusion, our findings suggest that while both Hibiscus and Pelargonium adjust their photosynthetic activity in response to varying CO_2_ levels, the mechanisms and efficiencies driving these responses differ significantly between the two species. These differences may have important implications for how each species adapts to environmental changes, particularly in the context of increasing atmospheric CO_2_ concentrations and climate change.

### 3.5. Fluorescence Data Reveal Distinct Mechanisms of Energy Use and Dissipation

The effective quantum yield of PSII (Fv’/Fm’) in Hibiscus was consistently lower than that in Pelargonium, which corresponded with higher non-photochemical quenching (NPQ) at lower PPFD levels (Figure 6). This suggests that Pelargonium may possess a more efficient electron transport mechanism under low-light conditions, which is critical for maintaining high photosynthetic efficiency under such scenarios [74]. The elevated NPQ in Hibiscus, particularly at lower PPFD, indicates that the species dissipates excess energy as heat to protect photosystem II (PSII) from photodamage [75,76]. This difference in NPQ might reflect variations in stress-response strategies between the two species or differing optimal light intensities for growth (Figure 2, Figure 4, Figure 5, Figure 6, Figure 10 and Figure 11; Table 1 and Table 2).

The consistently lower values of photochemical quenching (qP) and operational efficiency of PSII (ΦPSII) in Hibiscus across all PPFD levels suggest reduced energy conversion efficiency in its photosynthetic apparatus, which might explain the higher NPQ observed at lower light intensities. This could imply that Hibiscus invests more in non-photochemical pathways to avoid over-excitation of PSII, potentially at the expense of photochemical quenching [77].

### 3.6. Modifications to Chloroplast Ultrastructure

The JIP test provided key insights into the fluorescence parameters, highlighting distinct differences between Hibiscus and Pelargonium that can be attributed to their cellular and ultrastructural characteristics. Notably, Hibiscus exhibited significant increases in ϕ(PO), ϕ(EO), and PI_ABS_, which align with previous findings in plant species featuring densely stacked grana [78]. The Hibiscus ultrastructure, with tightly packed grana and reduced stromal space, likely enhances energy-trapping efficiency in its chloroplasts (Figure 10 and Figure 11) [79].

In contrast, Pelargonium demonstrated suboptimal photosynthetic efficiency, as indicated by its lower ϕ(PO) and ϕ(EO). This reduced performance is likely linked to the presence of less prominent chloroplasts with fewer thylakoids, corroborating earlier research that associates fewer thylakoids per chloroplast with lower photosynthetic capacity [80]. The lack of electrodensity in Pelargonium chloroplasts further supports the notion of compromised energy-conversion efficiency.

A notable feature of Pelargonium is the high abundance of mitochondria and unidentified cytoplasmic residues. This suggests a possible diversion of cellular resources towards metabolic pathways not directly related to photosynthesis, such as the production of defensive compounds, glandular trichomes, and volatile substances. This may represent a metabolic trade-off between maximising photosynthetic efficiency and allocating carbon resources to growth acceleration and defence mechanisms [71].

In Hibiscus, the ultrastructural observation of abundant oleaginous substances and mucilage indicates a well-developed system for the accumulation of metabolites. This aligns with the high values observed for SFI_ABS_ and PI_ABS_, supporting efficient conversion and storage of chemical energy. Conversely, despite Pelargonium’s high mitochondrial content, its lower Φ(RO) values and non-significant Ψ(RO) suggest that its chloroplasts are less optimised for photosynthesis. This structural limitation is reflected in the functional capacity of Pelargonium chloroplasts, which seem to be less adapted for energy efficiency [81].

### 3.7. Phenomenological Models

The JIP test results revealed a significantly higher RC/CS ratio in Hibiscus, suggesting a greater density of reaction centres per unit of chlorophyll. This supports the hypothesis that a higher concentration of reaction centres enhances the efficiency of energy utilisation in photosynthetic photon flux density (PPFD) [1,29]. The 10.91% increase in ABS/CS in Hibiscus further corroborates earlier findings, indicating that species with elevated absorption rates are more adept at converting absorbed light into electron transport in the PSII reaction centres [4].

In contrast, the observed decrease in DIo/CS for Hibiscus aligns with its lower NPQ values, supporting studies that suggest plants with reduced energy dissipation tend to utilise photochemistry more efficiently, driving higher electron transport rates (ETR) and consequently boosting ATP and NADPH production [4]. The elevated TRo/CS and ETo/CS values in Hibiscus indicate not only improved energy-trapping efficiency but also a faster electron transport rate, both of which are essential for accelerating the Calvin cycle and enhancing carbon fixation [82,83].

Conversely, Pelargonium demonstrated lower values for these parameters, which may indicate a prioritisation of alternative metabolic pathways. This aligns with the idea that plant species may adjust their energy utilisation strategies based on their evolutionary history and ecological niche [84,85,86]. Such metabolic trade-offs suggest that Pelargonium may be optimising for stress resistance or other ecological functions at the expense of photosynthetic efficiency.

The distinct photosynthetic efficiencies observed in these species can also be attributed to structural and biochemical variations in their leaves. For instance, Hibiscus leaves, characterised by their dark green hue and waxy epidermis, may be more effective at light absorption. In contrast, Pelargonium’s lighter green leaves with visible trichomes are associated with lower absorption and higher nonphotochemical quenching (NPQ) levels (Figure 6). These findings suggest that Hibiscus is better adapted for efficient photosynthesis, while Pelargonium may rely on protective adaptations, such as trichome density, to mitigate environmental stress [81].

In summary, the JIP test analysis reinforces the notion that Hibiscus has evolved a more optimised structure for photosynthesis and energy use, while Pelargonium has adapted alternative strategies, potentially prioritising defence mechanisms or other metabolic functions. These variations are likely reflective of their differing evolutionary pathways and ecological adaptations [87,88]. Further investigation is warranted to fully understand the species-specific mechanisms governing these photosynthetic and metabolic trade-offs.

### 3.8. Leaf Anatomy and Ultrastructural Morphology

Grounded in the biological principle that “form dictates function,” the observed differences in leaf parameters between Hibiscus and Pelargonium provide insights into how these species have adapted to various environmental conditions. For instance, the greater leaf thickness in Hibiscus, particularly in the palisade and spongy parenchyma, supports the hypothesis that thicker leaves are more efficient at absorbing incident light [35]. The contrast in trichome density between the two species likely reflects different strategies for defence against biotic stress or water conservation in response to abiotic factors. Trichomes have been shown to act as protective barriers against herbivores and help reduce water loss by thickening the boundary layer on leaf surfaces [42].

The higher density of chloroplasts in both the palisade and spongy parenchyma in Hibiscus may indicate a more efficient photosynthetic apparatus. This observation aligns with studies suggesting that increased chloroplast density optimises photosynthesis, particularly under low-light conditions, by enhancing light capture across the leaf profile [13,35,89]. The organisation of grana in Hibiscus, characterised by superdense stacking and reduced stromal regions relative to the lamellae, suggests more effective light-harvesting mechanisms. These features are consistent with the higher photosystem II efficiency and electron transport rates observed in the species, corroborating previous photosynthetic studies [13,35,64,89].

Additionally, the significant accumulation of plastoglobules in Hibiscus chloroplasts is notable. Plastoglobules, lipid–protein bodies located in the stroma, tend to accumulate under stress or during senescence [86,90]. Their presence in Hibiscus suggests a more robust oxidative stress response mechanism, allowing the plant to better adapt to fluctuating environmental conditions. In contrast, Pelargonium exhibited fewer thylakoids, with a lower electron density, which correlates with its reduced photosynthetic capacity and lower photosystem efficiency. The sparse presence of plastoglobules in Pelargonium suggests a less efficient oxidative stress management system [91].

Interestingly, the cytoplasm of Pelargonium was rich in unidentified residual substances, possibly indicating waste accumulation or an alternative metabolic pathway that is less dependent on chloroplast function for energy production. The high number of mitochondria adjacent to these sparse chloroplasts in Pelargonium may represent a compensatory mechanism. Mitochondria serve as major ATP production sites, particularly under suboptimal photosynthetic conditions [92], indicating that Pelargonium may rely more on mitochondrial energy production than on chloroplast-derived energy.

In Hibiscus, the presence of various oily substances in the cytoplasm is intriguing and suggests the accumulation of lipid droplets, which function as energy reserves during periods of stress [80,93]. These anatomical and ultrastructural observations further support the photosynthetic data, offering insights into the differential metabolic and photosynthetic adaptations of these two species. Such differences may help explain the varying ecological roles of Hibiscus and Pelargonium and their responses to environmental pressures (Figure 13).

## 4. Material and Methods

### 4.1. Environmental Conditions for Plant Growth

*Hibiscus rosa-sinensis* L. (commonly known as Hibiscus) and *Pelargonium zonale* (L.) L’Hér. Ex Aiton (commonly known as Pelargonium or Geranium) ornamental plants commonly used in horticulture. Both species naturally thrive in warm, humid environments with ample sunlight, conditions which were replicated in our greenhouse to ensure optimal growth. They were grown in 2 L pots filled with MecPlant^®^ (MecPrec Ind., Telêmaco Borba, Paraná, Brazil), a commercial substrate, and supplemented with NPK (10-10-10; 1 g pot^−1^) in a greenhouse under natural lighting conditions. Controlled temperature and humidity ranges of 22–26 °C and 60–70%, respectively, were maintained, along with a 13-hour light cycle with varying light intensity throughout the day. To standardise the water supply, the plants were watered at two specific times: once in the morning at 8 a.m. and once in the evening at 6 p.m. A total of 100 plants of each species were cultivated and analysed. For the purpose of this research, young, fully expanded leaves were collected and used for multiple analyses [94,95]. A combined total of 200 leaf samples were harvested for dual analyses: hyperspectral reflectance and leaf biochemical profiling [94,95]. To ensure consistency in data acquisition, all measurements were conducted between 11 a.m. and 1 p.m., except for specific adjustments made during measurement periods.

### 4.2. Spectral Characterisation of Leaf Optical Properties by Hyperspectral Analysis

The spectral attributes of leaf reflectance (R) and transmittance (T) were quantitatively assessed using a FieldSpec^®^ 3 spectroradiometer (Analytical Spectral Devices ASD Inc.). (Longmont, CO, USA). This instrument was interfaced with an ASD Contact PlantProbe^®^ with a 10 mm diameter. The spectroradiometer was equipped with 512 silicon photodiodes, enabling the capture of spectral data across the wavelength range of 350–2500 nm. To control for atmospheric interference, an ASD PlantProbe^®^ leaf clip was utilised during data collection, calibrated, and optimised using standard white Spectralon^®^ reference plates provided by Labsphere Inc. (Longmont, CO, USA) [35]. A strong light source from the PlantProbe^®^ was directed at the adaxial (upper) leaf surface [35]. Simultaneously, a second probe without an active light source assessed the abaxial (lower) leaf surface [35]. Reflectance (R) and transmittance (T) measurements were acquired simultaneously across diverse wavelengths. Each leaf sample was subjected to an average of 50 repeated measurements to construct a representative spectral curve. The absorptance (A) was subsequently calculated using the equation A = 1 − (R + T) [35]. This investigation integrated data corresponding to spectral curves and leaf pigments measured under a spectrophotometer by in vitro conditions, encompassing a wavelength range of 350 to 1100 nm.

### 4.3. Assessment of Leaf Tissue Composition

#### 4.3.1. Quantification of Chlorophyll and Carotenoids

A modified protocol described by Gitelson and Solovchenko (2018) [90] was used to quantify the concentrations of chlorophyll *a*, *b*, and *a*+*b*, in addition to carotenoids comprising both carotenes and xanthophylls for the apolar phase of the (2:1) chloroform/methanol extract. The absorbance of the methanol extract was measured at 470, 652, and 665 nm. Concentration calculations were performed based on Equations (1)–(4) proposed by Falcioni et al. (2017) [35], and the results are expressed in g m^−2^.

Chl*a* = 16.72 × Abs665 − 9.16 × Abs652 (1)

Chl*b* = 34.09 × Abs652 − 15.28 × Abs665 (2)

Chl*a*+*b* = Chl*a* + Chl*b* (3)

Car(C + X) = (1000 × Abs470 − 1.63 × Chl*a* − 104.96 × Chl*b*)/221 (4)

#### 4.3.2. Flavonoid and Anthocyanin Quantification

The polar fraction of the methanol extract (polar phase) was measured at λ358 nm for flavonoid assessment using molar absorption coefficients, as outlined by Gitelson and Solovchenko (2018) [90]. For anthocyanin quantification, the water–methanol phase was acidified with hydrochloric acid, and absorbance was measured at λ530 nm using a molar absorption coefficient referenced by Gitelson and Solovchenko (2018) [90].

#### 4.3.3. Analysis of Soluble Phenolic Compounds

The soluble phenolic compounds were quantified following a modified procedure from Ragaee (2006) [96]. An assay mixture consisting of methanolic extract, Folin–Ciocalteu reagent, Na_2_CO_3_, and deionised water was prepared, incubated in the dark, and subsequently centrifuged. The absorbance of the supernatant was recorded at λ725 nm. The equivalent PhC concentration was determined using gallic acid as a reference by regression Equation (5):

Ŷ = 83.432x + 1.8654; R^2^ = 0.993 (5)

#### 4.3.4. Assessment of Antioxidant Capacity

The total antioxidant activity was evaluated using a DPPH assay adapted from Llorach, Martínez-Sánchez, Tomás-Barberán, and Gil and Ferreres (2008) [56]. The reaction was initiated by adding 1 mM DPPH solution to the methanolic extract, with absorbance recorded following incubation by Equation (6).

% radical scavenging activity = (1 − (Abs_DPPH_/Abs_sample_) × 100) (6)

where Abs_DPPH_ = absorbance of DPPH, and Abs_sample_ = absorbance DPPH after 60 min.

### 4.4. Isolation of Protein-Free Cell Walls (PFCWs) and Lignin Quantification

A 150 mg sample of leaf powder underwent sequential washes and centrifugation, resulting in the isolation of protein-free cell walls (PFCWs) devoid of water-soluble compounds [97]. The lignin content of the isolated PFCWs was determined using the acetyl bromide method. Lignin concentration in the supernatant was determined using a standard curve.

### 4.5. Cellulose Quantification

Leaf samples were subjected to treatments with acetic/nitric acids and anthrone–sulfuric acid as described in Nagler, Inoue, Glenn, Russ and Daughtry 2003; Roig-Oliver et al. (2020) [98]. Cellulose concentrations were subsequently expressed in glucose equivalents.

### 4.6. Precision Assessment of Absorbance Profiles Via Optimal Wavelength Selection

To enhance the accuracy of discerning variations in chloroplast functionality and absorbance characteristics, we conducted analyses employing optimal wavelengths using hyperspectral bands. These computational/statistical analyses were facilitated by applying the normalised difference vegetation index (NDVI) as stipulated by Equation (7), conforming to the methodologies proposed by Crusiol et al. (2023) [99]. Each combination of the two spectral bands yielded a distinct hyperspectral vegetation index (HVI) upon the application of the NDVI algorithm. These unique HVIs were subsequently correlated with quantitative metrics indicative of the leaf’s optical properties. Custom-coded analyses were executed in the Interactive Data Language (IDL), utilising statistics including the Pearson correlation coefficient (r) and the coefficient of determination (R^2^). The sensor deployed for terrestrial measurements encompassed a spectral range of 350 nm to 1100 nm spectrophotometer analyses. Correlative findings were visualised as contour plots to facilitate interpretation.

(7) $$HVI=\frac{Wavelength\;1-Wavelength\;2}{Wavelength\;1+Wavelength\;2}$$

### 4.7. Gas Exchange Measurements

#### 4.7.1. Light Curves with Multiphase Flash^TM^ Fluorometer

The gas exchange measurements were performed on healthy, young, expanded leaves (the 5th or 6th leaf counting downwards from the apical meristem) of experimental leaves. An infrared gas exchange analyser (IRGA) (LI-6800, Li-Cor Inc., Lincoln, NE, USA) coupled with a Multiphase Flash^TM^ Fluorometer (LI-6800-01) was used to measure the net carbon assimilation rate (*A*), intercellular CO_2_ concentration (*C*_i_), stomatal conductance (*g*_s_), and transpiration rate (*E*). The photosynthetic light response curve was obtained using a manufacturer’s light source providing a range of photosynthetically active radiation (PPFD) [2500, 2000, 1800, 1500, 1200, 1000, 800, 600, 400, 300, 200, 150, 100, 75, 50, 25, and 0 µmol m^−2^ s^−1^] (the measurements in a major number of “points” promote better accuracy for estimating derived parameters). The analysis was performed under the following conditions: initial stabilised conditions for 20–30 min before star measurements, after which each point was obtained under the following conditions (110–150 s, min–max; red/blue ratio (90:10), constant 400 µmol mol^−1^ CO_2_ in the sample chamber, 60% relative humidity, medium flow rate of 700 µmol s^−1^ with ΔP (0.1) flow adjusted, VPD constant and automatised adjusted by Licor 6800, fan speed of 10,000 rpm, and temperature of 25 °C of leaf chamber. Fluorescence measurements were performed simultaneously with these readings.

The quantum yield of photosynthesis (α) [(µmol CO_2_ m^−2^ s^−1^)/(µmol photon m^−2^ s^−1^)], light compensation point (LCP) (µmol photons m^−2^ s^−1^), light saturation point (LSP) (µmol photons m^−2^ s^−1^), maximum net photosynthetic rate (*A*_MAX_) (µmol CO_2_ m^−2^ s^−1^) and dark respiration rates (Rd) (µmol CO_2_ m^−2^ s^−1^) were estimated using linear (*Y* = *ax* + *b*), hyperbolic models $\left[Y=y0+\frac{\left(ax\right)}{\left(b+x\right)}\right]$ or photosynthesis in relation to light and carbon dioxide $\left[PN=\frac{[\Phi \left(I0\right)\;\times \;I\;\times \;{\mathrm{Pg}}_{\mathrm{MAX}}]}{{{\left[\Phi (I0\right)}^{2}\;\times \;I^{2}+{{\mathrm{Pg}}_{\mathrm{MAX}}}^{2}]}^{0.5}}-Rd\right]$, where PN = net photosynthesis rate [mmol (CO_2_) m^−2^ s^−1^]; Φ(I0)= quantum yield at I = 0 [mmol (CO_2_) mmol^−1^ (photons)]; I = photosynthetic photon flux density [mmol (photons) m^−2^ s^−1^]; Pg_MAX_ = maximum gross photosynthesis rate [mmol (CO_2_) m^−2^ s^−1^]; RD = dark respiration rate [mmol (CO_2_) m^−2^ s^−1^]. In addition, the intrinsic water use efficiency (*i*WUE) was calculated using the relation *A*/*g*_s_ [(μmol m^−2^ s^−1^)/(mol m^−2^ s^−1^)] to consider the alterations resulting from leaf structures and ultrastructures in the photosynthetic curves.

#### 4.7.2. A−C_i_ Curves with Multiphase Flash^TM^ Fluorometer

Photosynthetic *A*−*C*_i_ response curves were also generated, and fluorescence measurements were performed simultaneously. Photosynthetic CO_2_ response (*A*−*C*_i_) curves were produced using CO_2_ chamber reference (CO_2__reference) concentrations [400, 300, 200, 100, 50, 25 400, 600, 800, 1000, 1200, 1400, 1600, 1800, 2000 µmol mol^−1^ and fixed light of 1000 µmol m^−2^ s^−1^ PPFD] using a commercial light source [(50–70 s, min–max; red/blue ratio (90:10)]; 60% sample chamber relative humidity (%RH_sample); flow 700 µmol s^−1^ with ΔP (0.1) for flow adjusts and VPD constant and automatised adjust by Licor 6800; fan speed 10,000 rpm; 25 °C heat exchanger temperature. These parameters were used to determine the carboxylation efficiency of the plants. The estimated rates of day respiration (Rd*; µmol CO_2_ m^−2^ s^−1^), maximum carboxylation rate of RuBisCO (*VC*_MAX_; µmol CO_2_ m^−2^ s^−1^), maximum rate of triose phosphate use (ΓCO_2_; µmol CO_2_ m^−2^ s^−1^), maximum rate of electron transport for the given light intensity (*J*_MAX_; µmol photons m^−2^ s^−1^), stomatal conductance (*g*_s_; µmol CO_2_ s^−1^ mmol^−1^), mesophyll conductance to CO^2^ transfer (*g*_m_; µmol CO_2_ s^−1^ mmol^−1^), chloroplast conductance to CO_2_ transfer (*C*c; µmol CO_2_ s^−1^ mmol^−1^), and electron transport in maximum chloroplast conductance to CO_2_ transfer (*A*_J_; µmol CO_2_ s^−1^ mmol^−1^) were calculated using the script “PCE_Calculator_Curve_Fitting_Model 2.0,” developed for tobacco plants and made available in “Plant Cell & Environment 2016” (Sharkey 2016) [24]. The constants for the equipment parameters were adjusted to a leaf temperature of 25 °C in the sample chamber, atmospheric pressure (Patm) of 101 kPa, and O_2_ concentration of 21 kPa [24].

#### 4.7.3. Daily Photosynthetic Measurements

Starting at 6 a.m. and concluding at 8 p.m., daily photosynthetic assessments were conducted using an infrared gas exchange analyser (IRGA) (LI-6800, LI-COR Inc., Lincoln, NE, USA) in conjunction with a Multiphase Flash™ Fluorometer (LI-6800-01). The apparatus was calibrated to measure net carbon assimilation rate (*A*), intercellular CO_2_ concentration (*C*_i_), stomatal conductance (*g*_s_), and transpiration rate (*E*). Measurements were performed at intervals with light settings configured at 1000 µmol m^−2^ s^−1^ (PPFD). The evaluations were performed under meticulously controlled conditions: a red/blue light proportion of 90:10, a consistent chamber CO_2_ concentration of 400 µmol mol^−1^, relative humidity stabilised at 60%, a medium flow rate set at 700 µmol s^−1^, a fan speed calibrated at 10,000 rpm, and an ambient temperature maintained at 25 °C. These measurements were in line with the protocols described in Section 4.7.1. We opted to use a standard, controlled condition instead of a “natural” light and temperature fluctuation during daylight. We collected comparable data throughout the day.

#### 4.7.4. Fluorescence Induction Kinetics

Chlorophyll a fluorescence induction kinetics (ChlF) data were collected using an LI-6800 IRGA (gas exchange system (LI-COR Inc., Lincoln, NE, USA)). Detached leaves were acclimated overnight in the dark in a humid chamber before data collection. Fluorescence curves were obtained using the following settings: 6 cm^2^ sample chamber, 75% relative humidity, 400 ppm CO_2_, fan speed of 10,000 rpm, pulse of saturating light (625 nm) of 15,000 µmol m^−2^ s^−1^ for 1 s, dark mode at 500 Hz, and flash mode rate at 250 kHz output rate by aligning at the induction mode measure. Each point obtained for relative fluorescence intensity at 20 µs, 50 µs, 100 µs, 300 µs, 2 ms, and 30 ms, as well as Fmt0 − tf, was used to calculate the JIP test parameters between 20 µs and 1 s. The curves were normalised to variable fluorescence (ΔVt), where t0 represents the initial time for fluorescence before the flash, tf denotes the final time for fluorescence after the flash, and the difference in kinetics for each OJIP phase was calculated using green leaves (white-light reference) as a reference, following Strasser et al. (2000) [30]. The five bands, ΔL (at ~20 µs), ΔK (at ~300 µs), ΔJ (at ~2 ms), ΔI (at ~10 ms), and ΔH (at ~40 ms), were calculated, resulting in 925 points of high-resolution curves. Each band corresponds to a specific phase of the energy transfer process (pastoquinones, plastocyanin, cytochrome b6f, and ferredoxin) within photosystem II (PSII) and photosystem I (PSI) during photosynthesis. Biolyzer software v4.0^®^ (Laboratory of Bioenergetics, University of Geneva, Geneva, Switzerland) was used to estimate the JIP test parameters associated with the electron transport chain of plants, according to Falcioni et al. (2024) [26]. The pipeline models of energy fluxes through the leaf RC−CSs were created using CorelDraw 2020^®^ (Corel Corp., Ottawa, ON, Canada) based on Sitko’s model [100].

#### 4.7.5. Fluorescence Measurements

Fluorescence measurements were performed using an LI-6800 (Li-Cor Inc.) equipped with a multiphase flash fluorometer (LI-6800-01). Plants were dark-acclimated for 12 h (overnight) to measure the “dark-acclimated” fluorescence PPFD parameters, initial fluorescence (Fo) and maximum fluorescence (Fm). Variable fluorescence (Fv) was calculated as Fv = Fm − Fo, enabling the calculation of the Fv/Fm ratio (maximum quantum yield of PSII in dark-adapted leaves). Additional chlorophyll fluorescence measurements were conducted using “light−acclimated leaves” during the analysis of light response curves. The multiphase flash fluorescence protocol (MPF) was applied with a saturating intensity of 15,000 µmol m^−2^ s^−1^, a dark modulation rate of 5 kHz, and a light modulation rate of 50 kHz for an optimal signal-to-noise ratio. The maximum Chl fluorescence (Fm’) was measured at 250 kHz during the saturating pulse, and fluorescence was detected at wavelengths greater than 700 nm (Li-Cor Inc.). The effective quantum yield of PSII (Fv’/Fm’), operational efficiency of photosystem II (ΦPSII; $ɸPSII=\frac{\left[Fm^{\prime }-Fs\right]}{Fm\prime }$, operational efficiency of photosystem II under CO2 (ΦCO2; $ɸCO2=\frac{\left[ACO2+Rd\right]}{PFD\;ob}\;$electron transport rate through photosystem II (ETR; ETR=ɸPSII×ABSleaf×actinic light×0.5 ) (µmol m^−2^ s^−1^), nonphotochemical quenching (NP; $NPQ=\frac{\left[Fm-Fm^{\prime }\right]}{Fm}$), photochemical dissipation quenching (qP; $qP=\frac{\left[Fm^{\prime }-Fs\right]}{Fm^{\prime }-Fo^{\prime }})$, and nonphotochemical dissipation quenching (qN; $qN=\frac{\left[Fm-Fm\prime \right]}{Fo-Fo^{\prime }})$ were estimated using Li-Cor software version 1 in tandem with gas exchange measurements by Baker (2008) [25].

### 4.8. Preparation and Microscopic Analysis

#### 4.8.1. Sample Preparation

For analyses using optical microscopy (OM), scanning electron microscopy (SEM), and transmission electron microscopy (TEM), leaf samples were dissected using a scalpel blade into cubic millimetres in a paraffin-coated Petri dish filled with a droplet of fixative solution to properly immerse the small fragments, avoid damage, and rapidly preserve the samples. The fixative solution consisted of a modified Karnovsky fixative solution, as described by Karnovsky (1965) [101]. The fixative comprised 2.5% glutaraldehyde and 2% paraformaldehyde, dissolved in 0.05 M cacodylate buffer at pH 7.2. Subsequently, six hours of postfixation was performed using a solution of 1% osmium tetroxide and 1.6% potassium ferrocyanide in an identical cacodylate buffer.

The specimens were then subjected to overnight block contrast with a 0.5% uranyl acetate solution. This was followed by a graded dehydration process utilising a series of acetone concentrations ranging from 30 to 100%, with three repeated cycles at the final concentration. A designated subset of these samples was set aside for additional SEM-specific procedures. The remaining samples were infiltrated and polymerised using Spurr low-viscosity epoxy resin. The prepared blocks were sectioned into semi-thin and ultrathin slices with thicknesses of 1 μm and 70 nm, respectively, using an MTX Powertome X ultramicrotome (Boeckeler Instruments RCM Products, Egham, UK). Both glass and diamond knives were employed for sectioning, corresponding to the varying thickness requirements. All reagents used in the sample preparation protocol were of electron microscopy grade and sourced from either Sigma (St. Louis, MO, USA) or EMS (Electron Microscopy Sciences, 1560 Industry Road, Hatfield, PA, USA).

#### 4.8.2. Optical Microscopy

For optical microscopy (OM) analyses, 1 μm thick leaf sections were stained with 1% toluidine blue solution in borax buffer. Staining was expedited by briefly heating the samples on a hot plate at 70 °C for 5 s. Observations were conducted using a Leica ICC50 optical microscope (Leica Microsystems, Wetzlar, Germany). Various anatomical metrics, including overall leaf thickness, dimensions of the palisade and spongy mesophyll layers, and thicknesses of both the adaxial and abaxial epidermal layers, were quantified. The ratio between spongy and palisade layers was also calculated. Dimensional analyses were performed using ImageJ software (Available online: https://imagej.nih.gov/ij (accessed on 1 October 2024), and contrast enhancement in false colours and quantitative measurements were performed using the Image–Pro–Plus^®^ version 4.5 software (Media Cybernetics Inc., Rockville, MD, USA).

#### 4.8.3. Scanning Electron Microscopy

Leaf samples were initially processed using a critical point drying (CPD) method facilitated by a CPD–030 device (Bal-Tec AG, Balzers, Liechtenstein). Subsequently, the samples were mounted and sputter-coated with gold at a current of 50 mA for 150 s using a MED010 Balzer evaporator (Bal–Tec AG, Balzers, Liechtenstein). Observations were performed using a Quanta 250 scanning electron microscope operating at either 15 kV or 20 kV (Thermo Fisher Scientific, FEI Company, Hillsboro, OR, USA). Digital images were generated using the integrated FEI software. This setup facilitated the assessment of stomatal density and size on both adaxial and abaxial leaf surfaces, as well as the detailed characterisation of leaf trichomes. Image–Pro–Plus^®^ version 4.5 software (Media Cybernetics Inc., Rockville, MD, USA) was used for both quantitative and qualitative data interpretation.

#### 4.8.4. Transmission Electron Microscopy

Ultrathin sections (60 nm or 70 nm) were prepared and positioned onto copper mesh grids with a 300-mesh rating. Contrast enhancement was achieved by contrast with 3% uranyl acetate for 30 min, followed by an additional 15 min with lead citrate in accordance with the protocol established by Reynolds (1963) [102]. Observations were performed using a JEOL JEM-1400 transmission electron microscope operating at 80 kV and equipped with a digital imaging system (Leica Microsystems Inc., Deerfield, IL, USA). This setup facilitated a comprehensive evaluation of cellular ultrastructures, including chloroplasts, thylakoid membranes, mitochondria, vacuoles, cytoplasmic components, and plastoglobules. Both quantitative and qualitative analyses were conducted using the Image-Pro Plus^®^ version 4.5 software (Media Cybernetics Inc., Rockville, MD, USA).

### 4.9. Univariate and Multivariate Analyses

The homogeneity of variance across all variables was assessed using Bartlett’s test, which eliminated the need for data transformation. Quantitative results were evaluated using paired *t*-test and reported as the mean ± standard error (SE). A significance level of *p* < 0.01 was established as the criterion for statistical significance. When applicable, Pearson’s correlation coefficient was used to examine the interrelationships between the variables. All univariate statistical analyses were conducted using Statistica^®^ 10.0 (StatSoft Inc., Tulsa, OK, USA), SigmaPlot^®^ 10.0 (Systat Software, Inc., San Jose, CA, USA), and the R statistical package (R Core Team, 2020).

Multivariate analysis of the dataset related to growth parameters was conducted using principal component analysis (PCA) in The Unscrambler X software, version 10.4 (CAMO Software, Oslo, Norway). A significance threshold of *p* < 0.01 was applied to ensure the robustness of the analysis. To avoid underfitting and overfitting, the optimal number of principal components was determined based on the first peak value of the cumulative explained variance, as indicated by Jolliffe et al. (2016) [103]. Furthermore, PCA was employed to form clusters between the two species and the vectors associated with each cluster for each component of each species [103]. This approach provides a comprehensive understanding of growth parameters and their relationships in Hibiscus and Pelargonium plants.

## 5. Concluding Remarks

This study provides a comprehensive comparison between Hibiscus and Pelargonium, aiming to simplify complex analyses of photosynthetic profiles and offer valuable insights into their respective adaptations. Our findings reveal distinct physiological and metabolic strategies that optimise photosynthetic efficiency in each species. Hibiscus demonstrates superior photosynthetic performance, which is supported by its robust chloroplast architecture and advantageous leaf anatomical features. These traits make Hibiscus highly efficient at trapping and utilising light energy. In contrast, Pelargonium appears to prioritise alternative metabolic pathways, possibly as part of a trade-off, as indicated by its higher mitochondrial content and less efficient chloroplast function.

These findings have important implications for agriculture, especially in selecting species for specific ecological roles or stress conditions. The study contributes to a deeper understanding of how structural features at the cellular level affect overall plant function, particularly in response to environmental conditions. By laying the groundwork for future research, this comparison underscores the need to further explore species-specific functional implications, which could have significant relevance in ecology, agriculture, and plant biology.

## Figures and Tables

**Table 1:** Estimated photosynthetic and fluorescence parameters in response to light and CO_2_ curves of Hibiscus and Pelargonium plants. The parameters include dark respiration rate (Rd; μmol CO_2_ m^−2^ s^−1^), light compensation point (LCP; μmol photons m^−2^ s^−1^), light saturating point (LSP; μmol photons m^−2^ s^−1^), maximum gross photosynthesis rate (*Pg*_MAX_; μmol CO_2_ m^−2^ s^−1^), maximum photosynthetic potential (*A*_MAX_; μmol CO_2_ m^−2^ s^−1^), and maximum quantum yield of photosynthesis (α; (μmol CO_2_ m^−2^ s^−1^)/(μmol photons m^−2^ s^−1^)), intrinsic water use efficiency (*i*WUE; (μmol CO_2_ m^−2^ s^−1^)/(μmol photons m^−2^ s^−1^)), day respiration (Rd*; μmol CO_2_ m^−2^ s^−1^), maximum carboxylation rate of RuBisCO (*VC*_MAX_; μmol CO_2_ m^−2^ s^−1^), maximum rate of triose phosphate use (ΓCO_2_; μmol CO_2_ m^−2^ s^−1^), maximum rate of electron transport for the given light intensity (*J*_MAX_; μmol CO_2_ m^−2^ s^−1^), stomatal conductance (*g*_s_; μmol CO_2_ m^−2^ s^−1^), mesophyll conductance to CO_2_ transfer (*g*_m_; μmol CO_2_ m^−2^ s^−1^), chloroplast conductance to CO_2_ transfer (*C*c; μmol CO_2_ m^−2^ s^−1^), and electron transport in maximum chloroplast conductance to CO_2_ transfer (*A*_J_), effective quantum yield of PSII (Fv’/Fm’), electron transport rate (ETR; μmol photons m^−2^ s^−1^), nonphotochemical quenching (NPQ), photochemical dissipation quenching (qP), nonphotochemical dissipation quenching (qN), and operational efficiency of photosystem II (ΦPSII). The estimated parameters for fluorescence were 400 μmol m^−2^ s^−1^. The Fv/Fm values for the maximum quantum yield of PSII in dark-adapted leaves are reported in Figure 5, Figure 6 and Figure 7 (inset) to be 0.87 and 0.86. The underlines indicate significant differences via the *t*-test (*p* < 0.01). Mean ± SE *(n* = 10).

Parameters		Species	
		Hibiscus	Pelargonium
**Photochemical**	Rd	1.10 ± 0.050	1.00 ± 0.020
	LCP	15.00 ± 0.440	18.00 ± 0.130
	LSP	322.00 ± 12.130	258.00 ± 5.460
	Pn_MAX_	10.60 ± 0.110	6.30 ± 0.150
	*A*_MAX_	9.60 ± 0.130	5.30 ± 0.150
	α	0.08 ± 0.003	0.06 ± 0.001
**Carboxilative**	*i*WUE	50.70 ± 1.100	89.00 ± 5.400
	Rd^*^	6.40 ± 0.660	4.00 ± 0.030
	*VC*_MAX_	24.30 ± 2.990	32.90 ± 1.470
	ΓCO_2_	1.90 ± 0.210	2.60 ± 0.160
	*J*_MAX_	52.30 ± 1.370	55.90 ± 1.790
	*g*_s_	0.10 ± 0.009	0.10 ± 0.003
	*g*_m_	9.60 ± 0.020	9.60 ± 0.010
	*C*c	10.10 ± 1.240	13.60 ± 0.610
	*A*_J_	10.50 ± 0.880	11.20 ± 0.360
**Fluorescence**	Fv’/Fm’	0.590 ± 0.006	0.59 ± 0.002
	ETR	53.63 ± 1.070	50.86 ± 0.250
	NPQ	1.30 ± 0.071	1.41 ± 0.019
	qP	0.59 ± 0.009	0.64 ± 0.003
	qN	0.61 ± 0.012	0.58 ± 0.005
	ΦPSII	0.38 ± 0.006	0.46 ± 0.002

**Table 2:** Estimated parameters of leaves in Hibiscus and Pelargonium plants. These included leaf thickness (LFT; μm), palisade thickness (PLT; μm), spongy leaf thickness (SPT; μm), adaxial leaf epidermis (AdLE; μm), abaxial leaf epidermis (AbLE; μm), spongy/palisade ratio (SPL ratio), adaxial stomatal density (ASD; n° mm^−2^), abaxial stomatal density (ABD; n° mm^−2^), stomatal size (Stoz; μm), adaxial tector trichomes (AdTT; n° mm^−2^), abaxial tector trichomes (AbTT; n° mm^−2^), adaxial glandular trichomes (AdGT; n° mm^−2^), abaxial glandular trichomes (AbGT; n° mm^−2^), palisade chloroplasts (PLC; n° cell^−1^), spongy chloroplasts (SPC; n° cell^−1^), and spongy/palisade chloroplast ratio (SPC ratio). Underlined parameters indicate significant differences by *t*-test (*p* < 0.01). “nd” means not detected. Mean ± SE (*n* = 10).

Parameters	Species	
	Hibiscus	Pelargonium
LFT	294.2 ± 3.61	280.2 ± 3.75
PLT	83.6 ± 1.88	71.7 ± 1.43
SPT	163.5 ± 2.92	153.6 ± 3.37
AdLE	29.5 ± 1.09	32.6 ± 0.94
AbLLE	17.5 ± 1.17	22.3 ± 0.90
SPL ratio	1.96 ± 0.06	2.15 ± 0.08
ASD	nd ± nd	39 ± 1.4
ABD	201 ± 3.6	101 ± 2.4
Stoz	25.1 ± 0.71	22.3 ± 0.35
AdTT	0 ± 0.0	4 ± 0.4
AbTT	2 ± 0.1	6 ± 0.4
AdGT	5 ± 0.3	11 ± 0.7
AbGT	10 ± 0.3	22 ± 0.6
PLC	14 ± 0.5	9 ± 0.4
SPC	7 ± 0.3	4 ± 0.2
SPC ratio	0.52 ± 0.03	0.42 ± 0.03

## Data Availability

Data are contained within the article.
